# SSRaDNet: An Empirical Study of Radar Object Detection Evaluation and Deployment Trade-Offs †

**DOI:** 10.3390/s26196074

**Published:** 2026-09-25

**Authors:** Iqbal Banwait, Niclas Zeller, Javad Alirezaie

**Affiliations:** 1 Department of Electrical, Computer and Biomedical Engineering, Toronto Metropolitan University, Toronto, ON M5B 2K3, Canada; iqbal.banwait@torontomu.ca; 2 Faculty of Electrical Engineering and Information Technology, Karlsruhe University of Applied Sciences, Moltkestraße 30, 76133 Karlsruhe, Germany; niclas.zeller@h-ka.de

**Keywords:** FMCW, ADC signal, object detection, segmentation, Swin Transformer

## Abstract

Millimeter-wave radar is an attractive sensing modality for autonomous driving because of its robustness under adverse weather and lighting conditions. However, comparisons between radar object detectors are hindered by inconsistent evaluation procedures and limited reporting of deployment costs. This work presents a comprehensive benchmark using standardized VOC mean average precision (mAP) and evaluates processing-pipeline latency across preprocessing, network inference, and post-processing. We evaluate SSRaDNet, a lightweight hybrid CNN–Swin Transformer detector, across analog-to-digital converter (ADC), range-Doppler (RD), and range-azimuth-Doppler (RAD) representations and compare it with existing methods on RADIal and RADDet. SSRaDNet achieves state-of-the-art AP@0.5 among re-evaluated RADIal models using RD input, reaching 88.89%, and provides the strongest overall balance of detection and segmentation performance among the compared ADC configurations. On RADDet, it achieves the best performance among methods using the native-resolution RAD tensor (45.69% mAP@0.5) and introduces the first ADC-based detector with full bounding-box regression. We also evaluate alternative post-processing strategies, including hybrid MaxPoolNMS–GreedyNMS, to characterize accuracy–latency trade-offs associated with alternative post-processing strategies. These results demonstrate the importance of standardized evaluation and full-pipeline runtime analysis for radar-based object detection.

## 1. Introduction

Radar sensors have become common components of advanced driver-assistance systems (ADASs) because of their relatively low cost and reliable operation under poor lighting and adverse weather conditions. They are widely used for applications such as blind-spot monitoring, adaptive cruise control, collision warning, and parking assistance. However, radar-based object detection remains less mature than camera and LiDAR-based perception. Conventional automotive radar often provides limited angular resolution, sparse object measurements, and little semantic information, making accurate localization and classification difficult. Higher-resolution radar systems can improve angular discrimination by using larger antenna arrays and collecting more measurements [1]. These improvements, however, increase data volume, memory requirements, and processing latency. The design of a practical radar perception system therefore requires a balance between sensing resolution, detection performance, and end-to-end execution speed.

Frequency-modulated continuous-wave (FMCW) radar is the most common radar technology used in automotive perception. These systems typically operate within the 76–81 GHz automotive band and transmit sequences of frequency-modulated signals known as chirps. The frequency difference between the transmitted and received signals provides range information, while phase changes across successive chirps are used to estimate radial velocity (Doppler). Multiple-input multiple-output (MIMO) radar systems use several transmitting and receiving antennas to synthesize a larger virtual antenna array [2]. For $N_{\mathrm{Tx}}$ transmitting and $N_{\mathrm{Rx}}$ receiving antennas, up to $N_{\mathrm{Tx}}N_{\mathrm{Rx}}$ virtual elements can be formed, depending on the array geometry. This approach provides an effective aperture comparable to a larger physical array while requiring fewer antenna elements and receiver channels. Phase differences across the virtual array enable azimuth estimation and, when supported by a two-dimensional antenna arrangement, elevation estimation. Increasing the number and spatial extent of the virtual elements generally improves angular resolution but also increases the volume of data that must be acquired, stored, and processed.

The sampled complex-valued measurements are referred to as analog-to-digital converter (ADC) data. Conventional FMCW processing applies a sequence of Fast Fourier Transforms (FFTs) to convert these measurements into interpretable radar representations. A range-FFT is first performed over the fast-time samples within each chirp. A Doppler-FFT is then applied across chirps for each range bin, producing a range-Doppler (RD) representation. Finally, angle processing is performed across the virtual antenna array for each range-Doppler cell, producing a range-azimuth-Doppler (RAD) tensor or an azimuth-elevation representation when a two-dimensional antenna array is available.

Each representation presents a different trade-off between retained information and computational cost. ADC data preserve the raw complex measurements and avoid fixed FFT-based preprocessing but require the network to learn useful spectral and spatial features directly. RD representations are more compact than full RAD tensors and require less preprocessing. When the antenna channels and their complex components are preserved, RD inputs can still contain information from which angular features may be learned [1]. In contrast, two-dimensional RD images formed by combining or discarding the antenna dimension contain little or no explicit angular information. RAD tensors provide explicit range, azimuth, and Doppler structure and often support strong detection performance, but their memory and processing requirements increase rapidly with sensor resolution. This trade-off makes full RAD processing particularly challenging for higher-resolution radar systems and motivates the investigation of ADC and antenna-preserving RD inputs. ADC, antenna-preserving RD, and RAD were therefore selected in this study to span different levels of radar signal processing and information retention, enabling comparison of their detection performance and deployment-related trade-offs under a common framework.

RAD tensors may be further converted into radar point clouds, producing a compact representation that is generally sparser and noisier than LiDAR point clouds. Radar point clouds are therefore frequently used in multi-modal perception systems, where camera or LiDAR data provide complementary spatial and semantic information [3,4,5,6]. Radar-only detectors more commonly operate on RAD [7,8], RD [1,9,10], RA [11,12], or multi-view representations [13,14,15,16].

Convolutional neural networks have historically formed the basis of many radar object-detection architectures, including ResNet-based and other two-dimensional designs [1,7,10,17]. Decourt et al. used compact RD images with a Faster R-CNN detector for radar-based object detection [10]. RadarResNet instead treats the range and azimuth dimensions of a RAD tensor as spatial coordinates and arranges Doppler bins along the channel dimension before applying a YOLO-style detection head [7]. FFTRadNet operates on antenna-preserving RD inputs and uses a learnable encoder to reduce channel redundancy before applying a PIXOR-style head for radar point detection [1].

Three-dimensional convolutional architectures have also been widely applied to radar object detection [13,14,17,18,19,20]. Several of these methods process multiple consecutive radar frames to exploit temporal information [13,14,18,21]. Although temporal context can improve detection performance, it may introduce non-causal inference when predictions for the current frame depend on future observations. Such designs are less suitable for real-time deployment unless the temporal input is limited to the current and preceding frames.

Compared with CNNs, Vision Transformers (ViTs) are well suited to capturing long-range dependencies and global context [22]. However, their computational cost and typically high data requirements have limited their adoption in radar perception. More efficient architectures, including Swin Transformers [23] and Retentive Vision Transformers [24], reduce these demands and have supported the development of competitive Transformer-based radar detectors [8,9,16,21,25]. Several radar models incorporate Transformer blocks in different ways. Giroux et al. demonstrated the effectiveness of pure Swin-based backbones on both lower- and higher-resolution radar datasets for point detection [9]. TransRSS adopts a hybrid encoder–decoder architecture that combines ResNet-based convolutional blocks with Transformer blocks in the bottleneck [16]. More recently, TransRAD employed a Retentive Vision Transformer backbone to improve radar feature modeling [8].

DANet introduced the Dimension Apart Module (DAM), which separates feature extraction across the range, azimuth, and temporal dimensions before concatenating the resulting features [20]. The module was designed to reduce parameter count and computational cost while preserving effective temporal feature extraction. T-RODNet later combined this dimension-specific strategy with Swin Transformer layers and introduced dilated convolutions in the spatial DAM branches to enlarge the receptive field [21]. These lightweight convolutional modules were placed before the Swin encoding stages, combining efficient local feature extraction with window-based self-attention.

Motivated by these approaches, this work builds on a series of developments toward the proposed SSRaDNet framework. Our first conference paper introduced the SCON block and combined it with a Swin Transformer backbone for radar point detection on RADDet [26]. A subsequent conference paper extended the detection head to full two-dimensional bounding-box regression on UWCR and evaluated single-frame point detection and freespace segmentation on RADIal using the original threshold-averaged evaluation protocol [27]. The present journal article substantially expands these studies through full bounding-box detection on RADDet, additional architectural ablations, conventional AP-based evaluation, standardized re-evaluation of publicly available RADIal models, input-footprint analysis, computational complexity analysis, and stage-wise latency and NMS evaluation.

Accordingly, the contributions of the present work comprise one architectural contribution and two complementary evaluation contributions:

1. Architecture and empirical validation: SSRaDNet (SCON-Swin Radar Detection Network), a hybrid CNN–Swin object-detection pipeline supporting ADC, RD, and RAD inputs across lower and higher-resolution radar datasets. Architectural ablations evaluate the contribution of the SCON block, while the resulting configurations achieve competitive or state-of-the-art performance within their respective input categories, including the strongest result among methods using RADDet’s native-resolution RAD tensor. In addition, SSRaDNet is the first ADC-based detector on RADDet to perform full bounding-box regression.
2. Standardized evaluation: A conventional AP-based re-evaluation of publicly available RADIal detectors, while retaining the original threshold-averaged metrics for comparison with methods that could not be independently reproduced.
3. Deployment-oriented evaluation: A stage-wise benchmark of radar preprocessing, neural-network inference, and detection post-processing under a common experimental setup, with complementary analyses of input footprint, computational complexity, and Greedy, MaxPool, and hybrid NMS accuracy–latency trade-offs.

## 2. Materials and Methods

The software implementation used in this study builds on the publicly available TFFTRadNet framework of Giroux et al. [9]. The following sections first describe the SSRaDNet architecture, followed by the evaluation and post-processing procedures used for standardized performance and computational comparisons. An overview of the SSRaDNet architecture and its various stages is shown in Figure 1.

### 2.1. Datasets

Several publicly available radar datasets support related perception tasks, but the combined evaluation considered in this work requires raw ADC measurements so that multiple radar representations can be generated and assessed under a controlled processing pipeline. This enables comparison of their explicit information, input memory footprint, and latency. RaDICaL provides measurements from a range of indoor and outdoor environments but includes relatively few object classes and limited Doppler information [28]. SCORP contains open-road segmentation annotations but does not support object detection [29]. UWCR provides low-resolution radar data for up to five classes [13], but many frames were collected in an empty parking lot, resulting in relatively simple driving scenes.

SSRaDNet was therefore evaluated on the complementary RADDet and RADIal datasets. RADDet [7] is a lower-resolution, multi-class dataset containing range-azimuth bounding boxes and class labels, enabling the evaluation of classification and full two-dimensional object detection. The dataset also exhibits substantial class imbalance. Bicycles, buses, and motorcycles account for only 3.3%, 0.8%, and 0.3% of the annotated objects, respectively, which is an important consideration when interpreting class-specific performance. In contrast, RADIal [1] is a higher-resolution dataset that provides vehicle-center annotations rather than variable-width bounding boxes and does not include class labels. It additionally provides freespace-segmentation annotations, allowing joint detection and segmentation performance to be assessed. Their operating environments also differ, with RADDet containing local-street and mixed-use scenes, while RADIal was collected primarily in freeway environments. Using two datasets that differ substantially in sensor configuration, annotation format, and driving environment allows us to assess whether SSRaDNet remains effective across distinct radar sensing regimes and perception tasks.

As summarized in Table 1, the two datasets were collected using substantially different radar systems. RADDet uses eight virtual antennas and a lower-resolution automotive radar configuration, whereas RADIal uses 192 virtual antennas, provides substantially finer azimuth resolution, and supports elevation estimation. Beyond the number of virtual antennas, radar performance can also depend on factors such as bandwidth, transmit power, propagation and scattering conditions, and the resulting signal-to-noise ratio. Because several of these parameters and conditions are not fully reported, they cannot be controlled across the public datasets. Absolute detection metrics are therefore not directly compared between RADDet and RADIal. Instead, results are interpreted within each dataset to assess the performance of SSRaDNet under different sensing configurations and tasks.

### 2.2. Preprocessing Techniques

The Fourier-Net preprocessing block follows a learnable spectral-transformation approach previously explored for radar-based human-motion recognition [30] and later applied to automotive radar point detection by Giroux et al. [9]. Rather than computing fixed Fourier transforms before inference, the block receives non-normalized ADC measurements and performs DFT-like transformations within the network. This allows the operation to be optimized jointly with the downstream detection objective. The transformation is implemented using complex-valued linear layers initialized from the DFT basis. For an input containing *N* samples, the complex weight associated with output bin *k* and input index *m* is initialized as

(1) $$w\left(k,m\right)=exp\left(-j\frac{2\pi }{N}km\right),\{\begin{array}{c} 0\leq k\leq M-1\\ 0\leq m\leq N-1 \end{array}$$

where *M* denotes the number of output frequency bins. The resulting transformation matrix therefore has dimensions M×N, reducing to a square N×N matrix when M=N. Tensor permutations are applied between the complex linear layers so that the range and Doppler-related dimensions are transformed in the appropriate order. Because the remainder of SSRaDNet operates on real-valued tensors, the real and imaginary components of the transformed output are separated and concatenated along the channel dimension. Instance normalization is then applied to produce a real-valued pseudo-RD representation while preserving both components of the complex response.

The initialized Fourier-Net block initially approximates conventional range and Doppler DFT operations. During end-to-end training, however, its weights are updated according to the detection and segmentation objectives rather than constrained to remain equal to the analytical DFT coefficients. The resulting transformation can therefore be interpreted as a learned perturbation of the DFT that generates pseudo-RD features adapted to the downstream task [30].

SSRaDNet also evaluates complex-RD and RAD representations. For RADIal, the precomputed antenna-preserving RD representations provided with the dataset are used directly. For the RADDet RD and RAD configurations, conventional FFT-based preprocessing is applied using a Hamming window. Following the Doppler FFT, fftshift is used to center the zero-Doppler bin, while zero-padding is applied before the azimuth FFT. For the RD configuration, the complex response from each antenna channel is retained, with its real and imaginary components arranged along the network channel dimension. This differs from a two-dimensional RD magnitude map, in which the antenna dimension is combined or discarded before network processing. The antenna-preserving complex RD representation therefore retains information from which angular features may be learned without explicitly constructing a RAD tensor. For RAD input, the Doppler dimension is instead used as the channel axis, and only the magnitude of the complex RAD response is retained.

### 2.3. SCON–Swin Backbone

The pseudo-RD tensor generated by Fourier-Net, or the RD and RAD tensors used when this block is bypassed, is processed by a hybrid CNN–Swin backbone derived from Swin-T [23]. The overall architecture, including the SCON block placement, is shown in Figure 1. Swin-T contains four hierarchical stages with 2, 2, 6, and 2 Transformer blocks, respectively. The input is first divided into 2×2 patches, after which patch-merging operations progressively reduce the spatial dimensions while increasing the channel capacity. This hierarchical structure allows features to be learned at multiple resolutions. Within each stage, Windowed Multi-Head Self-Attention operates over non-overlapping local windows, reducing the computational cost relative to global attention. Alternating shifted-window blocks then offset the window partitioning to enable information exchange between neighboring regions.

To introduce additional local feature extraction, our SCON block, detailed in Figure 2, is inserted before each Swin-T stage. The SCON block contains four parallel convolutional branches. The first two use 3×3 and 1×1 kernels, while the remaining branches use asymmetric 3×1 and 1×3 kernels to extract features independently along the two spatial dimensions. Each branch produces one-quarter of the input channel count. Unlike conventional spatially separable convolutions, the asymmetric kernels are applied in parallel rather than sequentially. The outputs of the four branches are concatenated with the original input and projected back to the required channel dimension using a final 1×1 convolution.

The contribution of the SCON block is examined through several backbone ablations. In one configuration, the SCON blocks are replaced with residual convolutional blocks to compare against a conventional CNN-based alternative. A dilated SCON variant is also evaluated, in which dilation is applied to the 3×1 and 1×3 branches. This design follows a principle similar to the dilated DAM module proposed by Jiang et al. [21], with the aim of increasing the receptive field while retaining dimension-specific processing. Finally, the convolutional blocks are removed entirely to evaluate the underlying Swin-T backbone without additional CNN-based feature extraction.

### 2.4. Decoder and Detection Head

The range-azimuth decoder converts the backbone features into an explicit RA representation for azimuth-based detection and segmentation. As the features pass through the backbone, their range resolution is progressively reduced while angular information is encoded across the channel dimension. Convolutional layers first adjust the feature dimensions, after which a tensor permutation maps the encoded angular features onto the azimuth axis. Transposed convolutions then restore the required range resolution, and final convolutional layers produce the task-specific RA feature maps.

The detection head follows the lightweight, single-stage design of PIXOR [31]. Predictions are generated over a discretized RA grid, with each cell representing a potential object center. The grid resolutions are 0.8m and $0.8^{\circ }$ for RADIal and 0.2m and $1.4^{\circ }$ for RADDet in the range and azimuth dimensions, respectively. To reduce the localization error introduced by this discretization, the network predicts continuous offsets relative to each grid-cell center. During ground-truth encoding, targets are assigned within a 3×3 neighborhood centered on each object, producing a localized heatmap around the corresponding grid cell.

Shared convolutional layers are followed by separate confidence, regression, and classification branches. The confidence branch produces a single-channel object-presence map and is optimized using focal loss to address the imbalance between foreground and background cells [32]. The regression branch predicts range and azimuth offsets for both datasets. For RADDet, it additionally estimates bounding-box width and height. Smooth $L_{1}$ loss is applied to these regression outputs. The classification branch is used only for RADDet and produces six class-specific prediction maps. RADIal contains a single object category and therefore does not require a separate classification output.

For RADIal, an additional freespace-segmentation head is included following the design introduced in [1]. Bilinear interpolation first adjusts the spatial resolution of the RA feature map, after which convolutional layers progressively reduce the channel dimension. A final 1×1 convolution produces the single-channel segmentation output. This head does not share its convolutional layers with the detection head, allowing it to learn specialized output features.

The network is trained using the weighted multi-task objective loss function:

(2) $$\begin{array}{cc} L(y_{b},y_{r},y_{c},y_{\mathrm{seg}})=\; & \alpha \;L_{\mathrm{Focal}}\left(y_{b},{\hat{y}}_{b}\right)+\beta \;L_{\mathrm{SmoothL}1}\left(y_{r},{\hat{y}}_{r}\right)\\ \; & +\gamma \;L_{\mathrm{CE}}\left(y_{c},{\hat{y}}_{c}\right)+\lambda \;L_{\mathrm{BCE}}\left(y_{\mathrm{seg}},{\hat{y}}_{\mathrm{seg}}\right) \end{array}$$

where *y* and $\hat{y}$ denote the corresponding ground-truth and predicted outputs. The weighting coefficients are set to α=2, $\beta =10^{2}$, $\gamma =10^{5}$, and $\lambda =10^{2}$. These values were chosen based on the optimization previously investigated by Giroux et al. [9]. The classification term is included only for RADDet, while the segmentation term is included only for RADIal.

### 2.5. Evaluation and Post-Processing Framework

#### 2.5.1. Non-Maximum Suppression

Unless otherwise stated, SSRaDNet results are reported using Greedy Non-Maximum Suppression (NMS) during post-processing. A confidence threshold of 0.05 is first applied during decoding. Greedy NMS is then performed independently for each class on RADDet and jointly across all predictions on RADIal. NMS IoU thresholds of 0.6 and 0.05 are used for RADDet and RADIal, respectively, based on validation-set performance. Because the SSRaDNet detection head produces heatmap-like prediction maps, it also supports the more parallelizable MaxPool NMS procedure, implemented using a kernel size of 5. MaxPool NMS is evaluated alongside a hybrid approach that first reduces the candidate set using MaxPool NMS and then applies Greedy NMS to refine the remaining predictions. These three approaches were selected to characterize the trade-off between the accuracy of conventional Greedy NMS and the latency enabled by the parallelized MaxPool-based suppression.

#### 2.5.2. Detection and Segmentation Evaluation

To provide a consistent object-detection evaluation across the RADDet and RADIal datasets, mean average precision is reported at IoU thresholds of 0.5 and 0.7 using a conventional confidence-ranked precision–recall evaluation procedure [33], which is widely used for object-detection benchmarking. For RADDet, the AP values are calculated independently for each object class and then averaged to obtain mAP. Since RADIal contains a single detection class, its reported mAP is numerically equivalent to AP.

The RADIal radar annotations and the outputs of the original point-based detection models specify vehicle locations without estimating variable bounding-box dimensions [1]. Following the original RADIal evaluation procedure, fixed-size bounding boxes are therefore assigned to both the predicted and ground-truth points before IoU-based matching. Consequently, the resulting AP primarily evaluates confidence ranking and center localization under a fixed box template rather than the accuracy of the estimated object extent. In addition to these metrics, the original RADIal evaluation protocol is retained for comparison with previously published results. For consistency with this protocol, precision and recall are first calculated independently at nine confidence thresholds:

(3) $$\tau_{i}=0.1i,\;i\in 1,\ldots ,9,$$

where $\tau_{i}$ denotes the *i*-th confidence threshold. At each threshold, precision and recall are calculated as

(4) $$P\left(\tau_{i}\right)=\frac{\mathrm{TP}(\tau_{i})}{\mathrm{TP}\left(\tau_{i}\right)+\mathrm{FP}\left(\tau_{i}\right)},$$

(5) $$R\left(\tau_{i}\right)=\frac{\mathrm{TP}(\tau_{i})}{\mathrm{TP}\left(\tau_{i}\right)+\mathrm{FN}\left(\tau_{i}\right)},$$

where $\mathrm{TP}(\tau_{i})$, $\mathrm{FP}(\tau_{i})$, and $\mathrm{FN}(\tau_{i})$ denote the numbers of true-positive, false-positive, and false-negative detections obtained after applying the confidence threshold $\tau_{i}$, respectively.

The reported precision and recall are then calculated by averaging the corresponding values across the nine confidence thresholds:

(6) $$\bar{P}=\frac{1}{N_{\tau }}\sum_{i=1}^{N_{\tau }}P\left(\tau_{i}\right),$$

(7) $$\bar{R}=\frac{1}{N_{\tau }}\sum_{i=1}^{N_{\tau }}R\left(\tau_{i}\right),$$

where $\left(N_{\tau }=9\right)$ is the number of evaluated confidence thresholds. Finally, the F1 score is calculated from the threshold-averaged precision and recall:

(8) $$F_{1}=\frac{2\bar{P}\times \bar{R}}{\bar{P}+\bar{R}}.$$

Although $\bar{P}$ and $\bar{R}$ were denoted as AP and AR in the original RADIal evaluation protocol, they are referred to as threshold-averaged precision and recall in this work. This terminology distinguishes them from conventional average precision, which is calculated from the area under a confidence-ranked precision–recall curve. In addition, free driving-space segmentation is also evaluated on the RADIal dataset using mIoU.

#### 2.5.3. Computational Complexity and Latency Evaluation

Computational complexity was characterized primarily using model parameter count, which provides a platform-independent measure that can be reported across a broader range of architectures. For the RADIal-based models that were independently re-evaluated using publicly available implementations, FLOPs were additionally estimated alongside latency. FLOPs were estimated using fvcore with a batch size of one and the native input dimensions of each model. Under the fvcore convention, one fused multiply-add is counted as one FLOP. Operations not supported by the default profiler, primarily elementwise activations and tensor operations, were not included.

Latency was measured separately for the major processing stages because radar input preparation, neural-network inference, and detection post-processing represent distinct components of the prediction pipeline whose computational costs may vary independently across input representations and architectures. Inference latency was measured using a batch size of one to represent single-frame processing. For each sample, raw ADC data were transferred to the GPU, after which the signal-processing stage (Fourier-Net or traditional FFTs) generated the required ADC, RD, or RAD-based representation. GPU events were used to separately measure the signal-processing and core neural-network inference stages, while wall-clock timing was used for post-processing, including output transfer to the CPU, detection decoding, and non-maximum suppression. The first 50 samples were excluded as warm-up iterations, and latency statistics were calculated over the remaining test samples, including the mean, median, and 95th percentile. Three DataLoader workers and pinned host memory were used to maintain consistent data delivery during evaluation.

Generative AI was used to assist with the development and review of selected evaluation scripts. All AI-assisted code was manually inspected, tested, and validated against the intended evaluation procedures before use.

## 3. Results

All models were trained and evaluated using a single NVIDIA GeForce RTX 4090 GPU with 24 GB of memory. Training was conducted for 150 epochs using batch sizes of 16 and 4 for the lower-resolution RADDet and higher-resolution RADIal datasets, respectively. The dataset partitions provided by the original authors were retained. RADDet used a 72/8/20 training, validation, and test split, while RADIal used a 70/15/15 split. Models were optimized using Adam [34] with an initial learning rate of $10^{-4}$, multiplied by 0.9 every 10 epochs.

To account for the performance variability observed primarily on RADDet, both datasets were evaluated at epochs 140, 145, and 150. Three independently trained models were assessed at each checkpoint, yielding nine checkpoint evaluations per configuration. Unless otherwise specified, reported values represent the mean across these nine evaluations.

### 3.1. RADDet Object Detection

Initial ablation experiments were conducted to evaluate the contribution of the proposed SCON block within the hybrid CNN–Swin backbone. The SCON-based architecture was compared with three alternative configurations: a conventional ResNet block, the removal of the CNN block to evaluate a pure Swin-T backbone, and a dilated SCON block employing dilated 3×1 and 1×3 convolutions. The dilated configuration was motivated by the improved performance reported for the DAM block proposed by Jiang et al. [21]. All configurations were trained using ADC inputs under the same experimental conditions, and the resulting detection performance is presented in Table 2.

The pure Swin-T backbone produced the lowest mAP at both IoU thresholds, indicating that the inclusion of a CNN-based feature-extraction block improved detection performance. This suggests that the local feature extraction provided by the convolutional component complements the window-based self-attention mechanism of Swin-T. The ResNet configuration provided a modest improvement over the pure Swin-T backbone but remained substantially below both SCON variants. The stronger performance of the SCON configurations relative to the ResNet configuration suggests that independently extracting features along the two spatial dimensions may provide representations that are more useful for downstream radar object detection. Relative to the pure Swin-T configuration, the standard and dilated SCON blocks improved mAP at an IoU threshold of 0.5 by 10.00 and 10.09 percentage points, respectively. At an IoU threshold of 0.7, the corresponding improvements were 4.63 and 5.33 percentage points. Although the dilated SCON block achieved the highest mean performance, its improvements over the standard SCON block were limited to 0.09 and 0.70 percentage points at IoU thresholds of 0.5 and 0.7, respectively. The standard SCON block also exhibited lower run-to-run variability, with standard deviations approximately 0.7 and 0.1 percentage points lower than those of the dilated configuration. Given the marginal performance advantage of dilation and the greater consistency of the standard configuration, the standard SCON block was selected for the subsequent experiments.

Table 3 compares the detection performance of the proposed SSRaDNet configurations with selected methods from the literature. To the best of our knowledge, the ADC-based configuration is the first approach evaluated on the RADDet dataset to perform two-dimensional radar object detection with full bounding-box regression directly from ADC data. It achieved mAP values of 41.40% and 18.89% at IoU thresholds of 0.5 and 0.7, respectively, although its performance remained below that of the RAD-based approaches. The SSRaDNet RAD configuration achieved performance comparable to RadarResNet at an IoU threshold of 0.5, exceeding it by 0.34 percentage points, while outperforming RadarResNet by 8.87 percentage points at an IoU threshold of 0.7. Among the compared methods, TransRAD achieved the highest overall mAP at both IoU thresholds. However, detection accuracy alone does not fully characterize deployment-related trade-offs, as the associated data and memory requirements can differ substantially. Table 4 therefore compares the nominal memory footprints of the input representations used by these methods.

Table 4 demonstrates that the choice of radar representation produces substantial differences in nominal input storage requirements. The SSRaDNet ADC input has dimensions (8×256×64) and is stored in complex64 format, resulting in a footprint of 1 MiB. Within the initial processing block, this input is converted into a (16×256×64) real-valued representation, preserving the nominal 1 MiB tensor footprint. Consequently, the ADC and complex RD configurations have comparable input and initial feature-map storage requirements, although their peak memory consumption may differ because of intermediate operations and temporary buffers. In comparison, the native (64×256×256) RAD tensor used by SSRaDNet and RadarResNet requires 16 MiB, representing a sixteenfold increase relative to the ADC and RD inputs. The larger (256×256×256) RAD tensor used by TransRAD requires 64 MiB, 4 times the footprint of the native RADDet tensor and 64 times that of the SSRaDNet ADC and RD inputs. DAROD has the smallest input footprint at 64 KiB because it constructs a (256×64) RD map by summing the RAD tensor over the angle dimension and processes the resulting map without resizing. This reduction substantially decreases the input-storage requirement during network inference but removes the angle dimension before feature extraction. These comparisons therefore illustrate a trade-off between tensor footprint and the amount of radar information retained by each input representation.

The class-specific precision–recall curves presented in Figure A1 of Appendix A provide further insight into the ADC-based configuration. Except for motorcycles, all classes attain a precision of 1.0 at low recall. However, none achieves full recall: most classes exceed 0.6 recall, while cars perform best, reaching beyond 0.8. The motorcycle curve begins at low precision, rises to approximately 0.6 at around 0.15 recall, and then declines sharply. The bus curve similarly exhibits a pronounced drop near 0.3 recall. Buses and motorcycles produce the weakest curves within their respective panels and are also the two least represented classes in the dataset, suggesting that their performance may be limited by the comparatively small number of available training examples.

Latency results were also collected for the RADDet dataset and can be seen in Table 5. The RD representation required the least DSP time, while the ADC and RAD configurations exhibited comparable preprocessing latency. This result is reasonable because the ADC pathway uses a learned transformation block to approximate the DFT, whereas the RD and RAD representations are generated using highly optimized FFT operations. Although RAD processing requires an additional angle FFT and produces a substantially larger tensor, its DSP latency remained comparable to that of the learned ADC transformation. The RAD configuration also achieved the lowest mean core-inference latency, followed by ADC and RD.

Previous experiments found that the RD configuration had a higher overall latency than the ADC configuration [9,26]. However, the stage-wise measurements in Table 5 show that this difference did not originate primarily from DSP. Instead, the largest contribution was post-processing, with Greedy NMS for RD requiring more than twice the mean time observed for ADC and RAD. Across independently trained models, the RD configuration consistently generated more prediction candidates and retained substantially more detections after NMS. This suggests that its longer post-processing time was not attributable only to overlapping duplicate predictions, since many candidates remained sufficiently distinct to survive suppression. The per-class Greedy NMS procedure was therefore required to process a larger candidate set and retain more final detections, increasing the number of required comparisons and the overall post-processing latency.

The RAD configuration achieved the lowest mean total latency, demonstrating that, under the evaluated GPU setup, a larger input representation does not necessarily result in slower processing. The upper-tail latency also differed considerably across representations. For RD, the total latency p95 was nearly twice its median, indicating substantially greater frame-to-frame variability, whereas RAD exhibited a tighter latency distribution. Post-processing accounted for approximately 20% and 22% of the mean total latency for ADC and RAD, respectively, compared with approximately 35% for RD. These findings show that post-processing can represent a substantial portion of the complete prediction pipeline and should be included in latency evaluations rather than reporting neural-network inference time alone.

Given the substantial contribution of post-processing to total inference latency, the choice of suppression method warrants explicit consideration. One advantage of the PIXOR-style detection head used in this work is its compatibility with Greedy, MaxPool, and hybrid NMS. As shown in Table 6, Greedy NMS consistently achieved the highest detection accuracy, whereas MaxPool NMS provided the lowest post-processing and total latency. For the ADC and RAD configurations, hybrid NMS retained most of the latency reduction provided by MaxPool NMS while recovering a considerable portion of its lost accuracy. Relative to Greedy NMS, the hybrid method reduced total latency by approximately 14.2% for ADC and 16.5% for RAD, compared with reductions of 15.4% and 17.7%, respectively, using MaxPool NMS. At the same time, hybrid NMS limited the reductions in mAP to 1.23 and 0.36 percentage points for ADC, and 1.17 and 0.22 percentage points for RAD, at IoU thresholds of 0.5 and 0.7, respectively.

The RD configuration exhibited a less favorable trade-off. MaxPool NMS reduced the post-processing share from 35.19% to 5.86% and decreased total latency by approximately 31.2% but also produced the largest accuracy reductions with 3.17 and 1.28 percentage points at IoU thresholds of 0.5 and 0.7. Hybrid NMS reduced these losses to 1.59 and 0.70 percentage points, although its post-processing latency remained approximately 3.6 times that of MaxPool NMS. This behavior is consistent with the larger number of prediction candidates and retained detections produced by the RD models, as discussed previously. Overall, hybrid NMS provides the most favorable latency–accuracy compromise for the ADC and RAD configurations, whereas the preferred RD post-processing method depends more strongly on whether deployment prioritizes accuracy or latency.

### 3.2. RADIal Point Detection

Table 7 compares radar-based point detectors on the RADIal dataset. Under the conventional AP evaluation, the RD-based SSRaDNet achieved the highest AP at an IoU threshold of 0.5, narrowly exceeding FFTRadNet. FFTRadNet performed substantially better under the stricter IoU threshold of 0.7, surpassing SSRaDNet by 9.63 percentage points. These results reveal a strength of FFTRadNet that is not apparent from the threshold-averaged precision and recall values reported in the original literature, particularly its ability to localize detections accurately under stricter IoU matching.

Under the original RADIal evaluation protocol, RD-based SSRaDNet increased recall by 7.0 percentage points relative to FFTRadNet while reducing precision by only 3.4 percentage points, resulting in a 2.4-point improvement in F1 score. It also achieved higher F1 scores than TFFTRadNet and CrossModal DNN. TransRSS reported the strongest threshold-averaged recall, F1 score, and mIoU, indicating the best overall performance under the original RADIal evaluation protocol. However, because its source code was unavailable, it could not be evaluated using the conventional AP procedure adopted in this work, preventing a complete comparison across both evaluation frameworks. Among the models evaluated under both the conventional AP and original RADIal protocols, RD-based SSRaDNet therefore provided the strongest overall balance between detection performance, recall, and freespace segmentation accuracy. Its mIoU was also the second highest overall, although the improvement over CrossModal DNN and RD-based TFFTRadNet was marginal.

The ADC-based SSRaDNet outperformed the ADC variant of TFFTRadNet in every reported metric except AP at an IoU threshold of 0.5, representing the strongest overall performance among the compared ADC-based configurations. Nevertheless, both ADC models remained below their corresponding RD variants across most metrics. This result suggests that directly processing ADC data may be less effective for the higher-resolution RADIal sensor configuration, consistent with observations from prior work [9].

Table 8 summarizes the parameter counts, FLOPs, and nominal input-memory requirements of the compared RADIal models. With the exception of TransRSS, the evaluated models use input tensors with the same nominal 16 MiB footprint. The model-complexity results also show that parameter count alone does not necessarily reflect computational cost. In particular, FFTRadNet has substantially fewer parameters than SSRaDNet and TFFTRadNet but requires the highest FLOP count among the models evaluated. This difference shows that parameter count and FLOPs capture distinct aspects of model complexity within the RADIal comparison.

TransRSS uses considerably smaller final RA and RD inputs, with nominal footprints of 1 MiB and 0.25 MiB, respectively. However, these views are generated from an intermediate (512×512×256) radar tensor, which would require 256 MiB if stored explicitly in float32 format. Its compact final inputs therefore do not necessarily reflect the full preprocessing and memory-bandwidth requirements of the pipeline, which depend on whether the intermediate tensor is explicitly stored or generated through streaming or dedicated radar-processing hardware.

The inference latency of SSRaDNet and the two comparison models with publicly available implementations was evaluated, as summarized in Table 9. All models were measured under the same conditions used for the RADDet experiments. As expected, the ADC configurations required greater DSP latency than their RD counterparts, consistent with the RADDet results. In contrast, NMS latency remained similar across all models, ranging from 0.97 to 1.21 ms. This limited variation suggests that the evaluated point detectors produced post-processing workloads of a comparable scale, potentially because all three models employ similar PIXOR-style detection heads.

FFTRadNet achieved the lowest overall latency, primarily due to its lower core-inference time, with a smaller advantage in NMS latency. Despite having the highest FLOP count among the re-evaluated models, it was the fastest, showing that operation count alone does not directly predict latency on a given hardware platform. Its total p95 latency of 16.14 ms was also close to its 14.67 ms median, indicating more consistent frame-to-frame execution than TFFTRadNet and SSRaDNet. TFFTRadNet was the second-fastest architecture for both input types while maintaining a favorable balance of latency, detection performance, and freespace segmentation accuracy. SSRaDNet required approximately 3 ms more total latency than the corresponding TFFTRadNet configurations, reflecting the added cost of its hybrid CNN–Swin architecture. Within each architecture, ADC and RD exhibited similar core-inference and total latency, unlike the larger input-dependent differences observed on RADDet.

The effects of Greedy and MaxPool NMS on all three models are compared in Table A1 of Appendix A. MaxPool NMS reduced post-processing latency for every model, although the reduction in total pipeline latency was modest because Greedy NMS already required only about 1 ms. SSRaDNet and FFTRadNet showed negligible AP changes, indicating faster post-processing without materially affecting detection performance. In contrast, TFFTRadNet showed AP reductions of 2.60 and 2.43 percentage points at IoU thresholds of 0.5 and 0.7, respectively.

Figure 3 presents representative detection and freespace-segmentation results produced from RD inputs. In the range-azimuth maps, the vertical axis spans the 103 m sensing range, while the horizontal axis covers the central $90^{\circ }$ of the radar’s $180^{\circ }$ azimuthal field of view. All predictions are generated in the radar coordinate system, while the bounding boxes shown in the camera images are included only for visualization and are obtained by projecting the radar detections into the camera frame.

## 4. Discussion

The findings of this study can be considered from two complementary perspectives. From an architectural perspective, the experiments evaluate SSRaDNet, which combines SCON-based local feature extraction, a Swin-T backbone, and an extended PIXOR-style detection head supporting full bounding-box regression across ADC, RD, and RAD representations. From an evaluation perspective, this study examines how metric definition, input representation, preprocessing, neural-network inference, and post-processing influence comparisons between radar detectors. Together, these results show that radar object-detection systems should be assessed in terms of both detection performance and the computational characteristics of the complete prediction pipeline.

### 4.1. Input Representation and Architecture

SSRaDNet combines convolutional feature extraction with a Swin-T backbone and was evaluated on the lower-resolution RADDet dataset and the higher-resolution RADIal dataset. The ablation results indicate that local convolutional processing complements window-based self-attention. The proposed SCON block applies 3×1 and 1×3 convolutions in parallel, allowing features to be extracted independently along the two spatial dimensions. While dimension-specific processing has previously been explored through DAM blocks in three-dimensional CNN architectures [20,21], the present results suggest that a similar principle is beneficial for two-dimensional radar representations when combined with a Swin-T backbone.

SSRaDNet was also evaluated using ADC, RD, and RAD inputs. The variety of radar representations used in the literature complicates direct comparison, particularly when their construction is not described precisely. For example, “RD input” may refer either to a tensor that preserves antenna information across its channels or to a two-dimensional RD image obtained after combining or discarding the antenna dimension. Although both representations may share the same name, they retain different information and have substantially different tensor footprints. Clearly reporting tensor dimensions, numerical precision, and preprocessing requirements is therefore important for both reproducibility and deployment-oriented comparison.

On RADDet, the RAD configuration achieved the strongest SSRaDNet detection performance, consistent with its explicit retention of range, azimuth, and Doppler information. However, the memory required by a RAD tensor increases rapidly with radar resolution and becomes increasingly difficult to scale when elevation is also included. This motivates the use of complex RD representations that preserve the virtual-antenna channels, allowing angular information to be learned without explicitly constructing a complete azimuth-elevation-Doppler representation [1]. Multi-view methods provide another alternative by processing compact RA and RD projections [13,16]. However, if these views are generated from a full radar tensor, the associated memory and bandwidth requirements are shifted to the preprocessing stage rather than eliminated. Overall, the results indicate that the preferred representation depends on the balance between retained radar information, sensor resolution, and available computational resources.

### 4.2. Evaluation and Deployment Trade-Offs

Conventional AP is widely used to evaluate object-detection models because it summarizes performance across the confidence-ranked precision–recall curve. In contrast, the original RADIal protocol averages precision and recall over several selected confidence thresholds. Although this approach enables comparison with earlier RADIal studies, referring to the resulting quantities as AP and AR may cause confusion because they differ from conventional object-detection metrics. Therefore, both evaluation protocols were reported in this work with a different name to preserve comparability with prior studies while also providing a standardized AP-based assessment.

Accurate reporting of input dimensions, numerical precision, and tensor footprint is similarly important for evaluation. Radar-detection studies use a wide range of ADC, RD, RA, RAD, and multi-view representations, and identical labels may describe tensors that retain different information. Input footprint therefore provides necessary context for both model comparison and deployment assessment. However, it should not be interpreted as total system memory usage, which also depends on preprocessing intermediates, model activations, temporary buffers, and hardware implementation.

To the best of our knowledge, this is the first study to report a stage-wise latency breakdown of radar DSP, neural-network inference, and detection post-processing under a common experimental setup across multiple radar input representations, radar datasets, and detection architectures. These measurements should be interpreted as relative processing-latency comparisons under a common RTX 4090 evaluation setup and do not establish execution time on embedded radar hardware or the achievable sensor update rate. The results demonstrate the importance of evaluating the complete prediction pipeline rather than considering preprocessing or neural-network inference in isolation. Although previous studies showed that learned Fourier-based transformations can reduce latency relative to conventional signal-processing pipelines [9,26], the present findings show that DSP latency alone does not determine total processing latency. Differences in core inference, prediction-candidate volume, and post-processing can offset or exceed preprocessing savings. For example, post-processing accounted for approximately 35% of the mean total latency of the SSRaDNet RD configuration. Reporting only neural-network inference time may therefore substantially underestimate complete processing-pipeline latency.

Many radar detectors employ YOLO or PIXOR-style prediction heads followed by Greedy NMS [1,7,8,9,11]. Greedy NMS performs iterative candidate selection and suppression, which limits parallelism and can become costly when a model produces many detections. MaxPool-based suppression can instead exploit parallel tensor operations, making it potentially attractive for hardware architectures that efficiently support such operations. In the present experiments, MaxPool NMS consistently reduced post-processing latency but generally produced lower detection accuracy, while hybrid NMS provided a more favorable compromise for several configurations. These findings indicate that post-processing should be treated as an architectural and deployment consideration rather than as a fixed implementation detail. Further work is needed to improve parallel suppression methods while preserving detection accuracy.

The stage-wise pipeline considered in this work could also be mapped onto heterogeneous automotive radar hardware. Conventional FFT-based preprocessing may be executed using dedicated radar accelerators, DSPs, or FPGA logic, while the learned Fourier-Net transformation and SSRaDNet backbone would require programmable linear-algebra or neural-network acceleration. Detection decoding and NMS could then be executed on a CPU/MCU or an accelerator, depending on the platform and suppression method. Modern automotive radar processors already combine several of these compute resources within a single device [35,36]. Accordingly, the stage-wise analysis presented here is intended to identify portions of the processing chain that may warrant optimization when porting the pipeline to representative automotive hardware.

### 4.3. Comparison with Prior Work

Several of the principal contributions of this study arise from its evaluation methodology rather than from the proposed architecture alone. These include the standardized AP-based re-evaluation of publicly available RADIal models and the stage-wise measurement of DSP, core inference, and post-processing latency across two datasets. Nevertheless, SSRaDNet remained competitive across several configurations. On RADDet, TransRAD achieved the strongest overall performance, exceeding SSRaDNet by approximately 9–10 percentage points at IoU thresholds of 0.5 and 0.7 [8]. This improvement was accompanied by a RAD input footprint four times larger than the native RADDet tensor used by SSRaDNet. Although full RAD representations can provide strong performance on lower-resolution sensors, their memory and bandwidth requirements increase rapidly with radar resolution. SSRaDNet instead supports ADC, RD, and RAD inputs, allowing the representation to be adapted to different sensing and hardware constraints. It also achieved the strongest result among the compared methods using the native RADDet tensor and, to the best of our knowledge, provided the first ADC-based detector on RADDet to perform full two-dimensional bounding-box regression.

TransRSS reported the strongest threshold-averaged recall, F1 score, and freespace-segmentation performance on RADIal [16]. Its final RA and RD network inputs are comparatively compact, although their generation may require access to a substantially larger intermediate radar tensor. The multi-view design also emphasizes projected RA and RD representations rather than preserving the complete antenna-domain information available in complex RD or ADC data. This may limit the direct extension of the approach to joint azimuth-elevation reasoning, which could be important for distinguishing elevated structures, such as bridges, from true freespace boundaries. However, the practical impact depends on the sensor geometry and preprocessing implementation. SSRaDNet did not surpass TransRSS under the original RADIal metrics, but it provided competitive multi-task performance while supporting minimally processed inputs and enabling evaluation under both the original and conventional AP protocols.

### 4.4. Limitations and Future Work

Several limitations affect the scope of this study. First, the absence of publicly available implementations for some RADIal models prevented their re-evaluation using conventional AP metrics and a common latency-measurement procedure. In addition, the calculation of the reported RADIal-style AP and AR metrics was not fully documented in some studies, so their direct comparability with the original evaluation protocol could not be independently confirmed.

The evaluated datasets were also collected using different radar hardware and acquisition configurations. Sensor-dependent factors such as transmit power, antenna configuration, bandwidth, propagation and scattering conditions, and received SNR can influence detection difficulty independently of the network architecture. Because several acquisition parameters are not publicly reported and the datasets were collected independently, these effects cannot be isolated retrospectively. Absolute detection performance is therefore interpreted within each dataset rather than directly compared across datasets.

All latency experiments were conducted on an NVIDIA RTX 4090 GPU, which differs substantially from the embedded hardware used in autonomous vehicles. Future work should therefore evaluate the complete pipeline on representative automotive computing platforms. Sensor acquisition must also be considered separately from processing latency. RADIal operates at 5 frames per second, corresponding to a 200 ms interval between measurements, which limits the effective update rate regardless of model speed. Improving radar acquisition rates and coordinating sensing with processing are therefore important system-level directions.

Finally, the availability and diversity of ADC radar datasets remain limited. The datasets used in this study were primarily collected during daylight and favorable weather, despite adverse conditions being a major motivation for radar sensing. RADDet also exhibits substantial class imbalance. In addition, few datasets bridge the gap between low-resolution, cost-effective sensors and high-resolution radar systems. Future dataset development should include diverse weather and lighting conditions, more balanced class distributions, raw ADC measurements, and intermediate-resolution radar configurations.

## Figures and Tables

**Figure 1:**
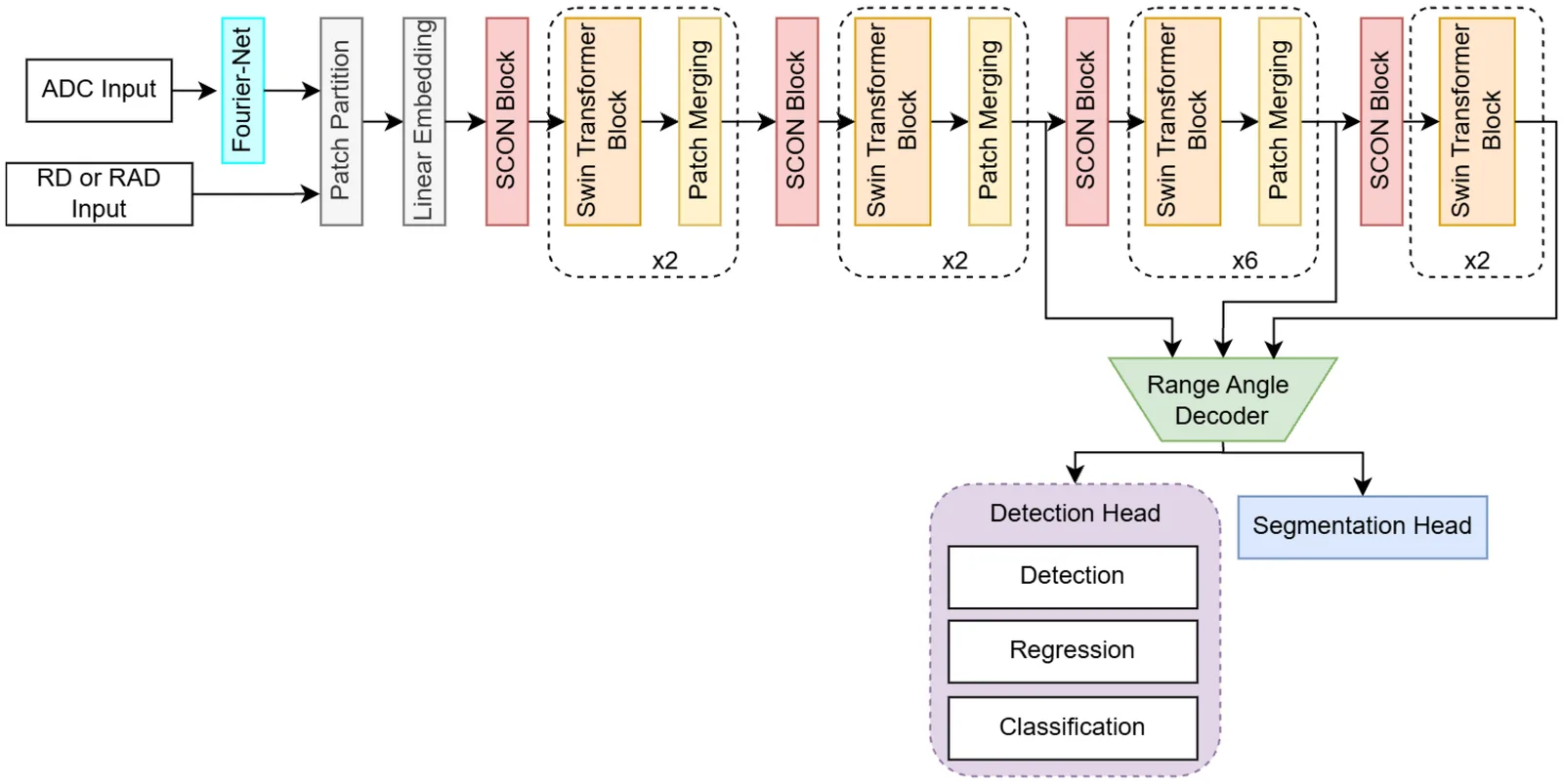
Overview of the SSRaDNet architecture. Model variants use ADC, RD, or RAD input. The segmentation head is included only for the RADIal dataset. The classification branch of the detection head generates class-specific binary prediction maps only for RADDet.

**Figure 2:**
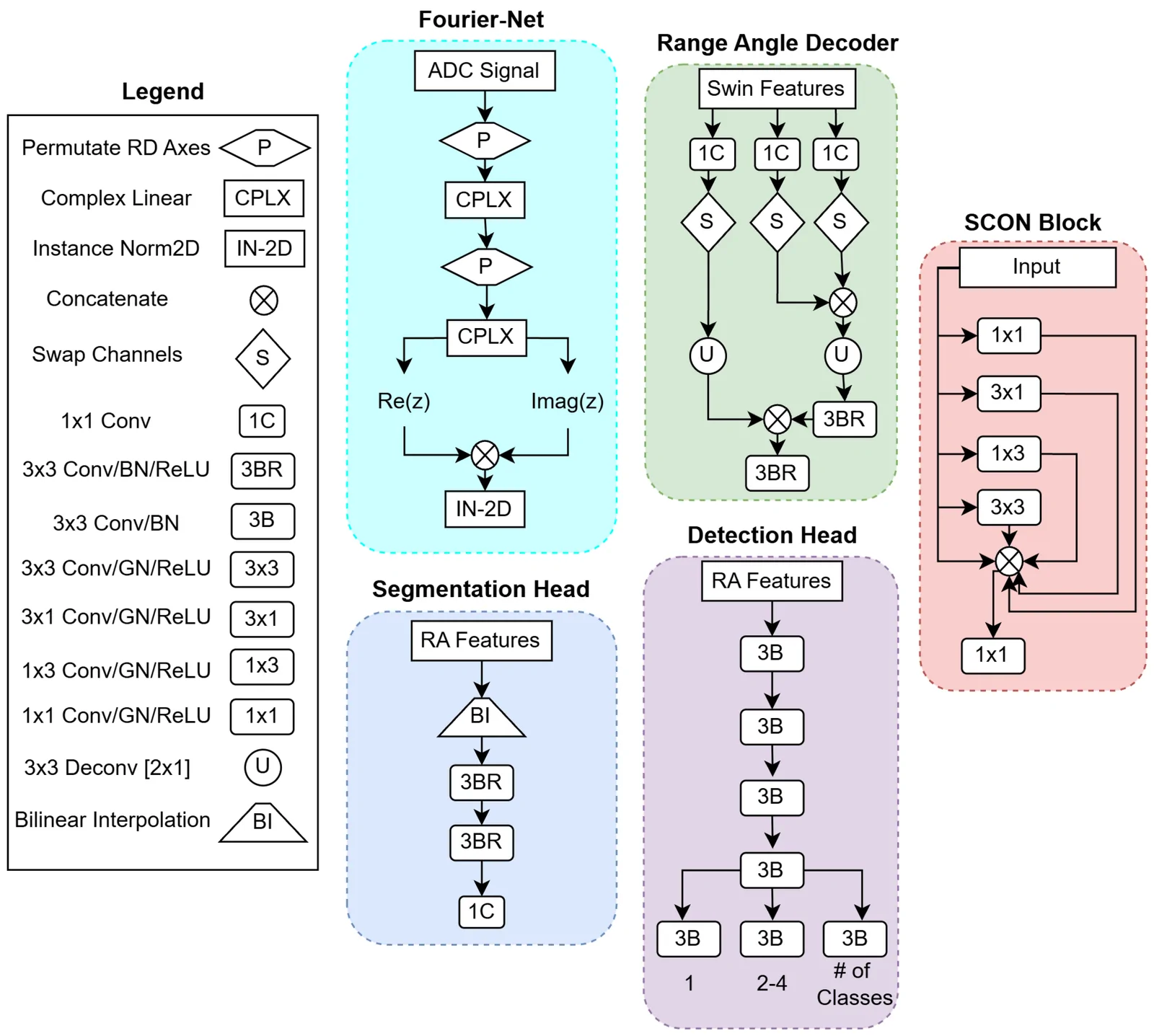
Detailed overview of the primary components and operations used in the SSRaDNet architecture. The number of classes is 6 for RADDet, and 0 for RADIal.

**Figure 3:**
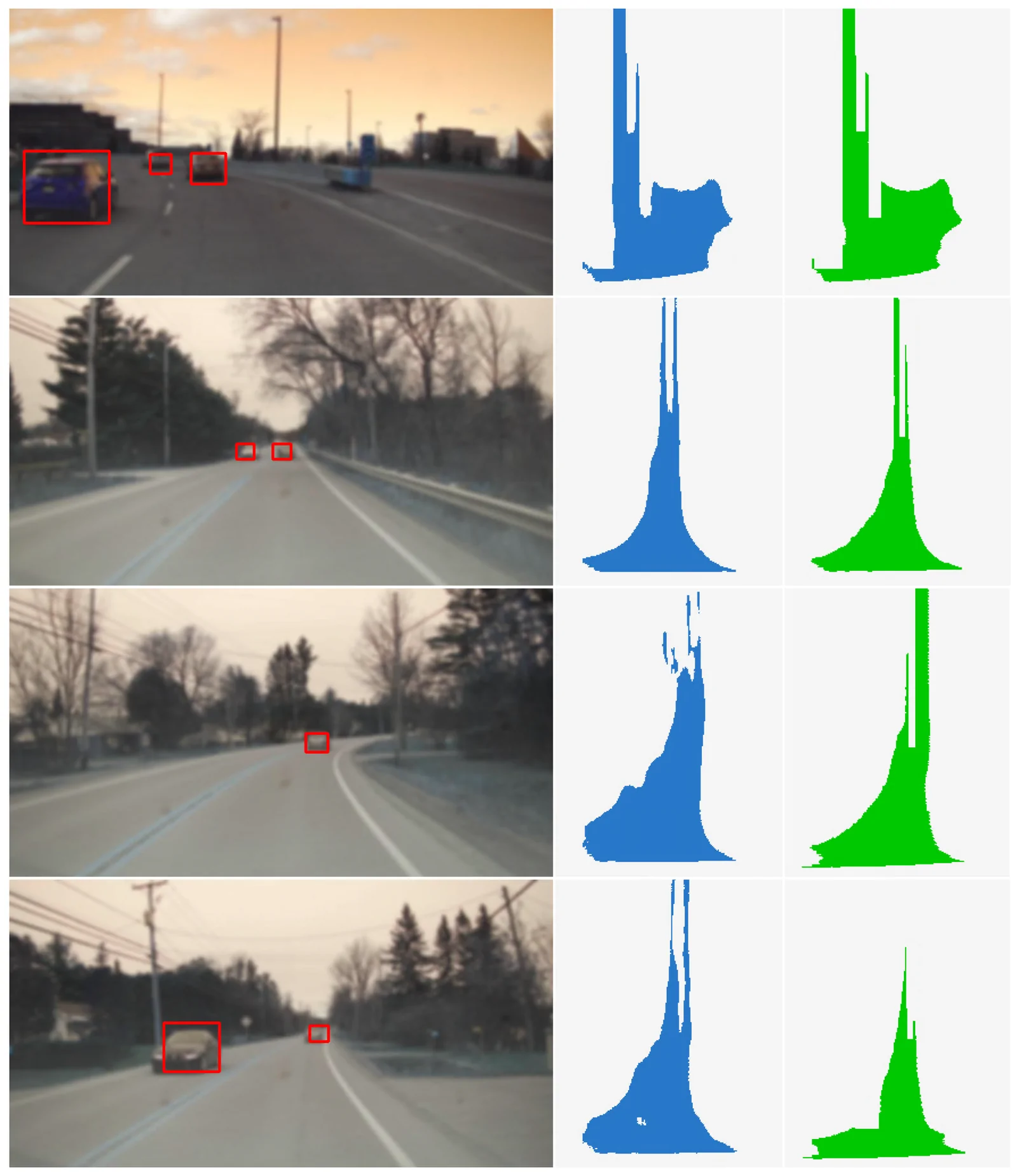
Representative RADIal results obtained using RD input. From left to right, the columns show camera images with projected radar detections (red boxes), predicted range-azimuth freespace maps (blue areas), and corresponding ground-truth freespace masks (green areas). Segmentation IoU values from top to bottom are 97.3%, 86.6%, 72.0%, and 61.3%.

**Table 1:** Comparison of radar sensor characteristics used in the RADDet and RADIal datasets.

Specification	RADDet	RADIal
Radar	TI AWR1843-BOOST	Custom cascaded HD radar
Configured frequency	77 GHz	Not reported
Hardware frequency range	76–81 GHz	Not reported
Acquisition bandwidth	Not reported	Not reported
Active Tx/Rx	2 Tx/4 Rx	12 Tx/16 Rx
Virtual antennas	8	192
Frame rate	Not reported	5 fps
Range resolution	0.1953 m	0.2 m
Maximum range	50 m	103 m
Azimuth resolution	≈$15^{\circ }$	$0.1^{\circ }$
Azimuth FOV	$180^{\circ }$	$180^{\circ }$
Elevation resolution	N/A ^†^	$1^{\circ }$
Elevation FOV	N/A ^†^	$12^{\circ }$

^†^ The AWR1843-BOOST hardware can support elevation estimation, but elevation is not represented in the RADDet RAD data used in this study. Parameters listed as “Not reported” were not specified for the dataset acquisition configuration in the publicly available dataset documentation.

**Table 2:** Effect of different CNN-based blocks on RADDet dataset detection performance. Results are reported as mean ± standard deviation.

Block Configuration	mAP at IoU = 0.5	mAP at IoU = 0.7
SCON block	41.40±0.91%	18.89±0.75%
ResNet block	34.62±2.20%	16.49±1.29%
No block	31.40±0.89%	14.26±6.20%
Dilated SCON block	41.49±1.64%	19.59±0.87%

**Table 3:** Comparison of SSRaDNet with selected radar object detection methods on the RADDet dataset.

Model	Input Type	mAP at IoU = 0.5	mAP at IoU = 0.7
SSRaDNet	ADC	41.40%	18.89%
SSRaDNet	Complex RD	41.78%	20.18%
SSRaDNet	RAD	45.69%	23.65%
RadarResNet * [7]	RAD	45.35%	14.78%
DAROD * [10]	RD	32.14%	10.86%
TransRAD * [8]	RAD	**55.90%**	**32.16%**

* Results for RadarResNet, DAROD, and TransRAD are reported by Cheng et al. [8]. Bold values indicate the best overall result, while underlined values indicate the second-best reported result.

**Table 4:** Parameter counts and nominal memory footprints of the radar input representations used by SSRaDNet and selected RADDet-based detection methods.

Representation	Parameters (M)	Tensor Dimensions	Numerical Precision	Tensor Footprint
SSRaDNet ADC	8.26	8×256×64	complex64 *	1 MiB
SSRaDNet RD	8.12	16×256×64	float32	1 MiB
DAROD RD [10]	N/A	256×64	float32	64 KiB
SSRaDNet RAD	8.85	64×256×256	float32	16 MiB
RadarResNet RAD [7]	9.59	64×256×256	float32	16 MiB
TransRAD RAD [8]	5.78	256×256×256	float32	64 MiB

* ADC inputs use complex64 precision and are subsequently represented as separate float32 real and imaginary components with the same nominal footprint. SSRaDNet and RadarResNet use the native RADDet RAD tensor dimensions. The DAROD and TransRAD inputs follow their respective implementations [8,10]. N/A indicates an unavailable parameter count. Tensor footprints refer only to the model input and not peak pipeline memory usage.

**Table 5:** Inference latency breakdown for the SSRaDNet input configurations on RADDet dataset.

Input Type	Processing Stage	Mean (ms)	Median (ms)	p95 (ms)
ADC	DSP	0.51	0.44	0.88
	Core inference	10.76	9.90	16.97
	Greedy NMS	2.76	2.52	4.79
	Total	14.04	13.00	20.84
RD	DSP	0.29	0.19	0.94
	Core inference	11.68	9.74	23.08
	Greedy NMS	6.50	6.32	9.77
	Total	18.46	16.79	30.89
RAD	DSP	0.50	0.48	0.64
	Core inference	9.66	9.19	13.05
	Greedy NMS	2.83	2.56	5.01
	Total	12.98	12.42	17.27

DSP denotes the input-specific digital signal processing stage, while p95 denotes the 95th-percentile latency. Core inference measures the latency of the neural-network forward pass. The Greedy NMS stage includes GPU-to-CPU transfer, NumPy conversion, standard detection decoding, and per-class greedy non-maximum suppression. Total latency is the sum of the DSP, core inference, and Greedy NMS stages.

**Table 6:** Effect of post-processing method on latency and detection performance for the SSRaDNet input configurations.

Input	Method	Post-Processing (ms)	Total (ms)	Post-Processing Share	mAP at IoU = 0.5	mAP at IoU = 0.7
ADC	Greedy NMS	2.76	14.04	19.69%	**41.40%**	**18.89%**
	Hybrid NMS	0.78	12.05	6.45%	40.17%	18.53%
	MaxPool NMS	**0.60**	**11.88**	**5.09%**	38.90%	17.92%
RD	Greedy NMS	6.50	18.46	35.19%	**41.78%**	**20.18%**
	Hybrid NMS	2.64	14.60	18.06%	40.19%	19.48%
	MaxPool NMS	**0.74**	**12.71**	**5.86%**	38.61%	18.90%
RAD	Greedy NMS	2.83	12.98	21.77%	**45.69%**	**23.65%**
	Hybrid NMS	0.68	10.84	6.27%	44.52%	23.43%
	MaxPool NMS	**0.53**	**10.68**	**4.92%**	44.08%	23.30%

Latency values represent mean per-frame measurements. Total latency includes input-specific DSP, core model inference, and the selected post-processing method. Post-processing share was calculated as the post-processing latency divided by the total latency. Bold values indicate the best result within each input configuration for the corresponding metric.

**Table 7:** Comparison of object detection and freespace segmentation performance on the RADIal dataset.

Model	AP@0.5	AP@0.7	Precision	Recall	F1 Score	mIoU
SSRaDNet (RD)	**88.89%**	64.13%	93.4%	89.2%	91.3%	80.60%
SSRaDNet (ADC)	86.75%	57.38%	90.7%	87.4%	89.0%	79.89%
FFTRadNet [1]	88.62%	**73.76%**	96.8%	82.2%	88.9%	73.98%
TFFTRadNet (RD) [9]	88.37%	61.79%	89.6%	89.5%	89.5%	80.19%
TFFTRadNet (ADC) [9]	87.13%	56.64%	88.2%	86.7%	87.4%	79.61%
CrossModal DNN * [17]	—	—	**96.9%**	83.5%	89.7%	80.40%
TransRSS * [16]	—	—	96.6%	**92.0%**	**94.2%**	**82.40%**

* Source code was not publicly available for CrossModal DNN or TransRSS, and therefore the AP could not be recalculated using the evaluation procedure adopted in this work. Precision and recall are averaged across confidence thresholds from 0.1 to 0.9, following the original RADIal protocol. The reported values for CrossModal DNN and TransRSS are assumed to use this procedure, although this could not be independently confirmed. Bold values indicate the best reported result, underlined values indicate the second-best reported result, and a dash indicates an unavailable metric.

**Table 8:** Model complexity and nominal input-memory requirements for radar-based models evaluated on the RADIal dataset.

Model	Parameters (M)	FLOPs (G)	Tensor Dimensions	Numerical Precision	Tensor Footprint
SSRaDNet (RD)	10.27	98.87	32×512×256	float32	16 MiB
SSRaDNet (ADC)	10.92	105.33	16×512×256	complex64 *	16 MiB
FFTRadNet [1]	3.79	144.22	32×512×256	float32	16 MiB
TFFTRadNet (RD) [9]	9.64	97.88	32×512×256	float32	16 MiB
TFFTRadNet (ADC) [9]	10.29	104.34	16×512×256	complex64 *	16 MiB
CrossModal DNN [17]	7.70	N/A	32×512×256	float32	16 MiB
TransRSS [16]	N/A	N/A	512×512 (RA) 256×256 (RD)	float32	1 MiB + 0.25 MiB

* ADC inputs use complex64 precision and are subsequently represented as separate float32 real and imaginary components with the same nominal footprint. N/A indicates that the corresponding value could not be reliably established.

**Table 9:** Inference latency breakdown for radar-based models on the RADIal dataset. All values are reported in milliseconds.

Model	Input	DSP Mean	Core Mean	NMS Mean	Total Mean	Total Median	Total p95
SSRaDNet	ADC	0.94	17.71	1.12	19.77	19.11	26.19
SSRaDNet	RD	0.39	18.01	1.15	19.56	18.51	25.91
TFFTRadNet	ADC	0.89	14.54	1.15	16.57	15.12	22.79
TFFTRadNet	RD	0.39	15.07	1.21	16.67	15.09	23.60
FFTRadNet	RD	0.39	13.90	0.97	**14.87**	**14.67**	**16.14**

DSP, core inference, and NMS columns report mean per-frame latency. Total latency includes all three stages. p95 denotes the 95th-percentile total latency. Measurements exclude data loading and radar acquisition (200 ms for RADIal setup). Bold values indicate the lowest total latency for each reported statistic.

**Figure A1:**
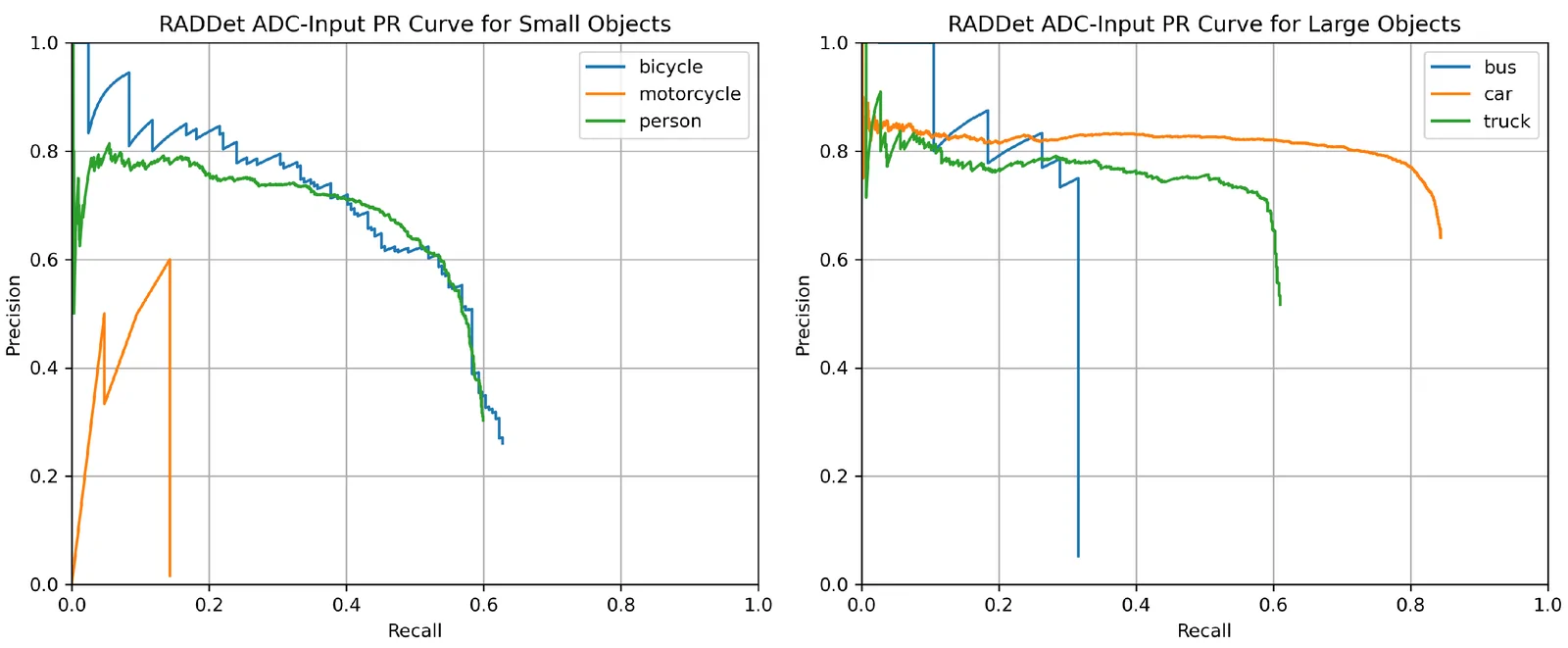
Class-specific precision–recall curves for the SSRaDNet ADC configuration on the RADDet dataset.

## Data Availability

The RADDet and RADIal datasets analyzed in this study are publicly available. RADDet is available at https://github.com/ZhangAoCanada/RADDet (accessed on 12 September 2024), and RADIal is available at https://github.com/valeoai/RADIal (accessed on 2 February 2025). The source code developed for this study is publicly available at https://github.com/IqbalBan/SSRadNet (accessed on 13 September 2026). No new datasets were generated in this study.

## Abbreviations

The following abbreviations are used in this manuscript:

ADAS	Advanced Driver-Assistance System
FMCW	Frequency-Modulated Continuous Wave
ADC	Analog-to-Digital Converter
RD	Range-Doppler
RA	Range-Azimuth
RAD	Range-Azimuth-Doppler
DSP	Digital Signal Processing
FFT	Fast Fourier Transform
DFT	Discrete Fourier Transform
NMS	Non-Maximum Suppression
CNN	Convolutional Neural Network

## Appendix A

**Table A1:** Effect of Greedy and MaxPool NMS on detection performance and post-processing latency for selected RADIal models.

NMS Method	Model	AP at IoU = 0.5	AP at IoU = 0.7	F1 Score	Post-Processing Latency (ms)
Greedy	SSRaDNet RD	**88.89%**	64.13%	**91.25%**	1.15
	FFTRadNet	88.62%	**73.76%**	88.91%	**0.97**
	TFFTRadNet RD	88.37%	61.79%	89.50%	1.21
MaxPool	SSRaDNet RD	**88.89%**	64.19%	**91.21%**	0.76
	FFTRadNet	88.61%	**73.89%**	88.87%	0.74
	TFFTRadNet RD	85.77%	59.36%	88.69%	**0.73**

Latency values represent mean per-frame post-processing times. Bold values indicate the best result within each NMS configuration for the corresponding metric.
